# Correlation of Structure and Electrocatalytic Performance of Bulk Oxides for Water Electrolysis

**DOI:** 10.3390/molecules30112391

**Published:** 2025-05-30

**Authors:** Chuanhui Zhu, Changming Zhao, Hao Tian, Shuk-Yin Tong

**Affiliations:** 1 School of Science and Engineering, The Chinese University of Hong Kong, Shenzhen 518172, China; 2 Institute of Materials Science and Devices, Suzhou University of Science and Technology, Suzhou 215009, China

**Keywords:** bulk oxides, water electrolysis, intrinsic activity, crystal structure, electronic structure

## Abstract

Hydrogen-centered electrochemical technologies play a vital role in sustainable energy conversion and storage. One of the challenges in achieving cheap hydrogen is to bridge the gap between advanced electrocatalysts and highly effective electrodes. The key lies in designing electrocatalysts with high intrinsic activity and understanding the structure–activity relationship in water electrolysis. Being proposed as promising electrocatalysts, bulk oxides, with their compositional and crystal structure flexibility, provide a good platform for studying the correlation between intrinsic activity and electronic structure and also for screening superior catalysts for water electrolysis. In this review, we discuss the recent developments of oxide electrocatalysts in understanding the structure–activity relationship. Firstly, we present a thorough overview of recent advances from both theoretical and experimental aspects. Subsequently, we highlight the design principles to provide guidance for promoting performance. Finally, the remaining challenges and perspectives about this field are presented. This review aims to provide guidance for the design of highly advanced oxide electrocatalysts for water electrolysis and large-scale green energy supply.

## 1. Introduction

With the growing demand for decarbonizing the industrial sector, increasing the share of clean and sustainable energy resources has become one of the top priorities for the science community in the coming decades. Therein, hydrogen economy coupling with water electrolysis has emerged as a key path to develop sustainable energy [1,2,3]. However, the high cost resulting from sluggish kinetics largely limits water electrolysis market penetration. The introduction of an electrocatalyst can effectively reduce the activation barrier, thus reducing the applied voltage and energy consumption of the reaction. To achieve low overpotential of the water electrolysis, it is of great urgency to develop electrocatalysts with excellent performance. To date, the widely used electrocatalysts for water electrocatalysis are dominated by noble metal electrocatalysts due to the significantly reduced overpotentials. However, the large-scale application of noble metal electrocatalysts is impeded by the rare reserves on earth and concomitantly high cost [4,5,6]. Therefore, the design of low-cost catalysts has been the research focus. Great attention, for example, was dedicated to developing earth-abundant transition metal-based nanomaterials, aiming to achieve high catalytic performance at a low cost. While the widely studied nanocatalysts surpass the commercial catalysts in the three-electrode configuration at laboratories, their performance can hardly be transferred to commercial electrolyzers operated in industrial conditions. Additionally, the nanocatalysts typically suffer from unsatisfactory repeatability, especially from the lack of a scaled-up synthesis strategy for industrial manufacture, and the instability for electrode fabrication of water electrolyzer techniques. For example, fabricating the electrode in alkaline water electrolyzers (AWEs) is generally prepared by platting the powder catalyst on the nickel net at an elevated temperature (plasma spraying), during which process the catalytic activity stemming from the quantum size effect, facet-dependent effect, and other nanostructured morphology benefits are largely removed. Therefore, research efforts on catalyst design are still urgently demanded.

Bulk oxides synthesized using a solid-state reaction demonstrate approachable scaled-up synthesis and are stable under industrial plasma spraying [7]. Especially, the advances in solid-state chemistry have facilitated the synthesis of numerous new oxides with enriched chemical space. The diversity of bulk oxides affords numerous degrees of freedom to tailor the physicochemical properties for enhancing the catalytic activity [8,9,10]. Here, the term ‘bulk oxides’ refers to materials that can be distinguished from nanoscale materials by their ability to be synthesized in large quantities via simple and cost-effective solid-state reactions, resulting in micrometer-sized particles as opposed to the more complex and expensive methods required for nanoscale oxides. In particular, bulk oxides address some critical aspects of water electrolysis (especially scaled-up synthesis using solid-state reactions), although they still have inherent drawbacks, such as low electronic conductivity and severely limited performance by virtue of the finite active sites in the bulk materials [11,12,13]. Therefore, bulk oxide electrocatalysts have emerged as promising electrocatalyst candidates to possess a highly efficient water electrolysis performance. More importantly, advancements in solid-state chemistry have facilitated the discovery of numerous oxides, and over 90% of the elements in the periodic table can be incorporated into the compositions and structures, such as perovskite oxides (*AB*O_3_), spinel oxides (*AB*_2_O_4_), and pyrochlore oxides (*A*_2_*B*_2_O_7_). Thus, bulk oxides with a flexible composition and crystal structure provide a good platform for studying the correlation between the intrinsic activity and electronic structure and screening for superior catalysts for water electrolysis [14,15,16]. In-depth understanding and significant progress have been achieved for bulk oxides in water electrolysis, such as the *e*_g_-filling descriptor, emphasizing the great potential of bulk oxides for achieving hydrogen economy. Focusing on the merits and disadvantages of bulk oxides in catalyzing water splitting, the need to review this exciting research field is motivated by the urgent need to unveil highly active electrocatalysts and intensify the systematic understanding of the structure–activity correlations.

In this review, we present a focused review of the correlation of the structure and electrocatalytic performance of bulk oxides for water electrolysis and summarize the rational guidelines for advanced electrocatalyst design. Firstly, we briefly introduce the recent developments in the design of highly efficient oxide electrocatalysts based on comprehensive theoretical and experimental advancements. Then, we introduce several rational strategies for catalyst design, including regulating the electronic structure and/or increasing active sites on the oxide electrocatalysts. Finally, we present the perspectives and challenges for the development of highly advanced oxide electrocatalysts for water electrolysis.

## 2. The Introduction of Water Electrolysis

### 2.1. Fundamental Concepts of Water Electrolysis

As a key technology for hydrogen production, water electrolysis typically operates within an electrolyzer, which consists of an electrolyte, cathode, and anode [17]. Under an applied voltage, water molecules would decompose into hydrogen and oxygen at the surface of electrodes, as shown in the following reaction:

H_2_O → H_2_ + 1/2O_2_

Based on electron transfer, the total reaction can be divided into two half-reactions: a hydrogen evolution reaction (HER) at the cathode and oxygen evolution reaction (OER) at the anode, where hydrogen and oxygen are generated at the respective electrodes. In addition, the half-reactions process varies depending on the electrolyte environment:

In acidic electrolytes:

Cathode: 2H^+^ + 2e^−^ → H_2_

Anode: H_2_O → 2H^+^ + 2e^−^ + 1/2O_2_

In alkaline and neutral electrolytes:

Cathode: 2H_2_O + 2e^−^ → H_2_ + 2OH^−^

Anode: 2OH^−^ → 1/2O_2_ + H_2_O + 2e^−^

**Figure 1:**
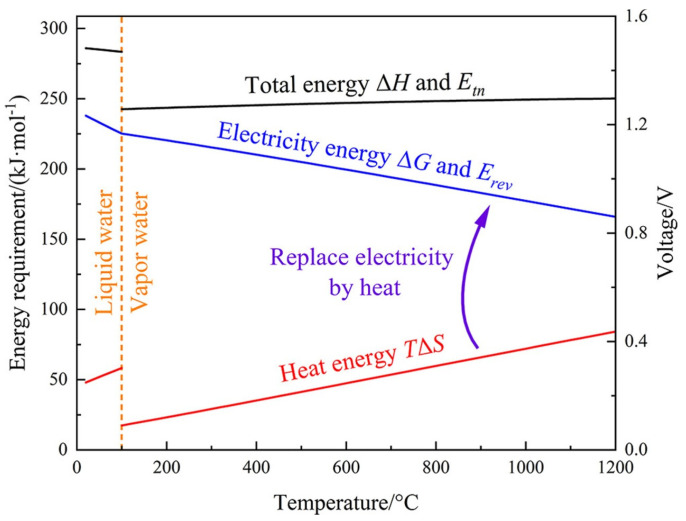
Thermodynamics of water electrolysis at atmospheric pressure [18].

Water electrolysis is an endothermic reaction, where the theoretical reversible (*E*_r_) and thermoneutral (*E*_t_) voltage are 1.229 and 1.481 V (at 25 °C, 1 atm), respectively. As shown in Figure 1, the *E*_r_ and *E*_t_ depend on the temperature, where the sudden change at 100 °C can be ascribed to the evaporation of water [18]. In addition, in practical applications, a much higher voltage than the *E*_r_ is required to bypass the activation barriers at the cathode (*η*_c_) and anode (*η*_a_), as well as various overpotentials (*η*_v_) caused by other factors, such as mass transport and other resistance. Thus, the overall working voltage (*E*_w_) is expressed as follows:

*E*_w_ = *E*_r_ + *η*_c_ + *η*_a_ + *η*_v_

Therefore, the actual working voltage for water electrolysis is usually over 1.6 V. Here, the overpotentials *η*_c_ and *η*_a_ arising from the slow reaction kinetics are largely dependent on the intrinsic activity of electrocatalysts as well as the structure of electrodes. To achieve efficient production of green hydrogen, developing highly efficient electrocatalysts for practical devices, such as alkaline water electrolyzers (AWE) and proton exchange membrane water electrolyzers (PEMWE), is critical for achieving hydrogen-based economies with zero-carbon emissions.

### 2.2. Oxygen Evolution Reaction and Hydrogen Evolution Reaction

As a critical half-reaction in water electrolysis, the OER occurs at the anode and involves a four-electron transfer process, resulting in more sluggish kinetics compared with the HER [18]. With respect to the OER mechanism, it is widely accepted that the OER can proceed through two typical mechanisms: the adsorbate evolution mechanism (AEM) and lattice oxygen-mediated mechanism (LOM) [19,20,21]. Currently, the AEM is the most commonly used OER mechanism, and the reaction steps in the different electrolyte environments are shown below:

In alkaline electrolytes:

OH^−^ + * → HO* + e^−^

HO* + OH^−^ → H_2_O + O* + e^−^

O* + OH^−^ → HOO* + e^−^

HOO* + OH^−^ → O_2_ + * + H_2_O + e^−^

In acidic or neutral electrolytes:

H_2_O + * → HO* + e^−^ + H^+^

HO* → O* + e^−^ + H^+^

O* + H_2_O → HOO* + e^−^ + H^+^

HOO* → O_2_ + * + e^−^ + H^+^

Here, * represents the active site (generally metal site for AEM) at the surface. With the increase in covalency of electrocatalysts, the binding energy of metal and oxygen intermediates would become weak; consequently, the LOM process for the OER may become favorable. In general, the lattice oxygens on the electrocatalyst surface would participate in the LOM-mediated OER process, which involves different lattice oxygen-associated pathways and nonconcerted proton–electron transfer. Therefore, the LOM-dominated electrocatalysts can overcome the adsorption scaling relation (HO* and HOO*) and the limitations of the minimum theoretical overpotential for the AEM process (~0.37 V) [22]. Nevertheless, the participation of lattice oxygen inevitably leads to the decline in stability, which needs to be resolved for the catalyst design [23]. In addition, the intrinsic origin of the LOM has not been fully understood, such as the bindings between lattice oxygen and oxygen intermediates are still in dispute [24]. Recently, more specific OER mechanisms have been proposed for the understanding of the OER process, such as the coupled oxygen evolution mechanism (COM) and oxide path mechanism (OPM) [25,26]. Understanding the OER mechanism can guide the development of more efficient OER electrocatalysts.

The HER occurs at the surface of the cathode, where the mechanism is associated with the adsorption/desorption of hydrogen intermediates on the catalyst surface, involving a two-electron transfer process: (1) the Volmer step; (2) the Heyrovsky or Tafel step (depending on the reaction pathway) [27,28,29]. The reaction intermediates and pathways vary depending on the pH:

In acidic electrolytes:

H^+^ + * + e^−^ → H* (Volmer)

H* + H^+^ + e^−^ → H_2_ + * (Heyrovsky) or 2H* → H_2_ + 2* (Tafel)

In alkaline or neutral electrolytes:

H_2_O + * + e^−^ → H* + OH^−^

H_2_O + H* + e^−^ → H_2_ + * + OH^−^ (Heyrovsky) or 2H* → H_2_ + 2* (Tafel)

The rate-determining step of the HER process can be identified using the Tafel slope (*b*) from the polarization curve. In general, if *b* < 30 mV dec^−1^, the Tafel step is the rate-determining step; if 40 mV dec^−1^ < *b* < 120 mV dec^−1^, the Heyrovsky step is the rate-determining step; and if *b* > 120 mV dec^−1^, the Volmer step is the rate-determining step. More importantly, in alkaline or neutral electrolytes, the energy barrier of water dissociation is a prerequisite in the HER process, resulting in higher activation barriers and slower kinetics. Therefore, a larger overpotential is needed to drive the reaction in the alkaline or neutral electrolytes. In addition, alkaline HER is the simplest cathodic reaction, involving only water dissociation and a two-electron transfer process. This makes it an important model reaction for investigating other complex reduction reactions, such as CO_2_ reduction and N_2_ reduction [30,31].

### 2.3. Performance Evaluation Parameters

The performance of water electrolysis is determined by both the surface kinetics and thermodynamics of the catalyst. To evaluate the catalytic activity of a given electrocatalyst, several key electrochemical parameters have been established to elucidate the overall performance of electrocatalysts, including the overpotential, Tafel slope, mass activity, specific activity, turnover frequency, faradaic efficiency, and stability [32]:

1. **Overpotential (*η*)**. The overpotential for water electrolysis is the extra applied voltage relative to the theoretical voltage under standard conditions, which is the commonly used parameter to assess the activity of the given electrocatalyst. Typically, the overpotential at *j*_geo_ (the geometric current density normalized to the electrode surface area) = 10 mA/cm^2^ (corresponding to a solar-to-hydrogen efficiency of 12.3%) is used as a key parameter to evaluate the catalytic activity [33]. However, the overpotential of the electrocatalysts is strongly affected by the specific surface area and loading mass, especially for nanocatalysts with large specific surface areas, which hinders the use of the overpotential to reveal the intrinsic catalytic activity [34].
2. **Tafel Slope (*b*) and Exchange Current Density (*j*_0_).** The two parameters can be obtained from the Tafel equation, *η* = a + *b*log *j*, where *b* is the Tafel slope, *j* is the current density, and *η* is the overpotential, respectively. Extrapolating the linear part of the Tafel plot to zero overpotential gives the exchange current density (*j*_0_), which reflects the intrinsic catalytic activity of the catalyst. Generally, an excellent electrocatalyst should have low *b* and high *j*_0_ values [35].
3. **Mass Activity (MA) and Specific Activity (SA)**. To further characterize the intrinsic activity of the catalyst, additional parameters, such as the mass activity (A/g) and specific activity, have been proposed based on the electrocatalyst mass loading, specific surface area, and electrochemically active surface area (ECSA). Mass activity is the current normalized by the current based on the catalyst mass loading. For electrocatalysts in equal mass, higher mass activity indicates greater catalytic efficiency, making it a useful parameter for assessing cost efficiency [36]. Specific activity is obtained by normalizing the current with the specific surface area or ECSA of the electrocatalysts, providing a more accurate reflection of intrinsic catalytic differences and facilitating the understanding of structure–activity relationships [37].
4. **Turnover Frequency (TOF), Faradaic Efficiency (FE), and Stability**. Turnover frequency represents the number of product molecules generated per active site per unit of time, making it a key parameter for evaluating the intrinsic catalytic activity of the electrocatalysts [38]. Faradaic efficiency is defined as the ratio of the experimental to the theoretical product, indicating the efficiency and selectivity of the electrocatalysts [39]. In general, the FE of efficient electrocatalysts for water electrocatalysis is expected to be close to 100%. In addition to the above parameters, the stability of electrocatalysts, including both performance stability and structural stability, is the key indicator to assess the application potential of the electrocatalyst [40]. Performance stability is usually assessed by long-term operating electrocatalytic measurements, such as cyclic voltammetry (CV), chronopotentiometry (CP), and chronoamperometry (CA) tests. Structural stability requires in situ or post-reaction characterizations to evaluate the changes in the composition, structure, and morphology of the electrocatalysts.

## 3. Overview of Bulk Oxides for Water Electrolysis

### 3.1. Overview of Bulk Oxide Electrocatalysts for Oxygen Evolution Reaction

**Figure 2:**
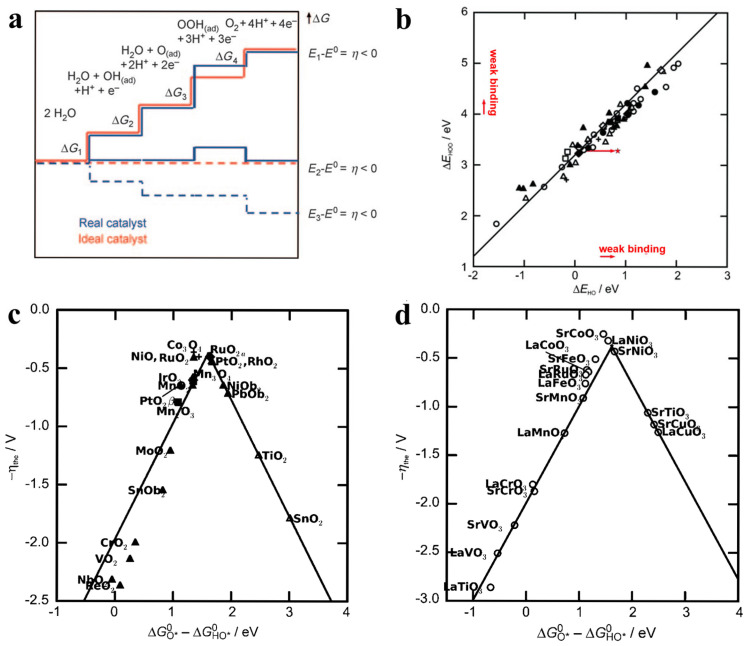
(**a**) Plot of Gibbs free energy of reactive species and intermediates (horizontal lines) of the oxygen evolution reaction (OER) versus the reaction coordinate [44]. (**b**) The adsorption energy of HOO* plotted against the adsorption energy of HO* on perovskites. (**c**,**d**) Volcano plot correlating OER catalytic activity with Δ*G*_O*_–Δ*G*_HO*_ [45].

Bulk oxides with flexible crystal structures and compositions provide excellent templates for investigating the structure–activity correlations in the OER [16,41,42]. Based on the widely accepted OER catalytic mechanisms, Nørskov et al. proposed a standard hydrogen electrode (SHE) computational model to simulate free energy changes during the OER process, which has greatly enhanced the understanding of OER mechanisms [43]. Later, Nørskov et al. investigated the relationship between the adsorption energy of reaction intermediates and the OER activity of the electrocatalysts [44,45]. For an ideal OER electrocatalyst, the free energy change (Δ*G*) for each reaction step is 1.23 V, resulting in an overall Δ*G* of 4.92 V (Figure 2a). However, in the actual OER processes, the free energy changes for different reaction steps are not identical, where the slowest step determines the overall reaction rate. More theoretical and experimental results indicate the adsorption energy of reaction intermediates can reflect the OER activity of a catalyst, such as Δ*G*_O*_ and Δ*G*_O*_–Δ*G*_HO*_ (Figure 2b–d). Therein, the electrocatalyst with oxygen intermediates with a binding energy that is neither too strong nor too weak exhibits the optimal activity. Experimentally, RuO_2_ with moderate adsorption energy exhibits the best OER activity. In addition, the volcano plot is also applicable for predicting the performance of other transition metal-based systems, making it one of the most widely accepted and mature descriptors to date.

**Figure 3:**
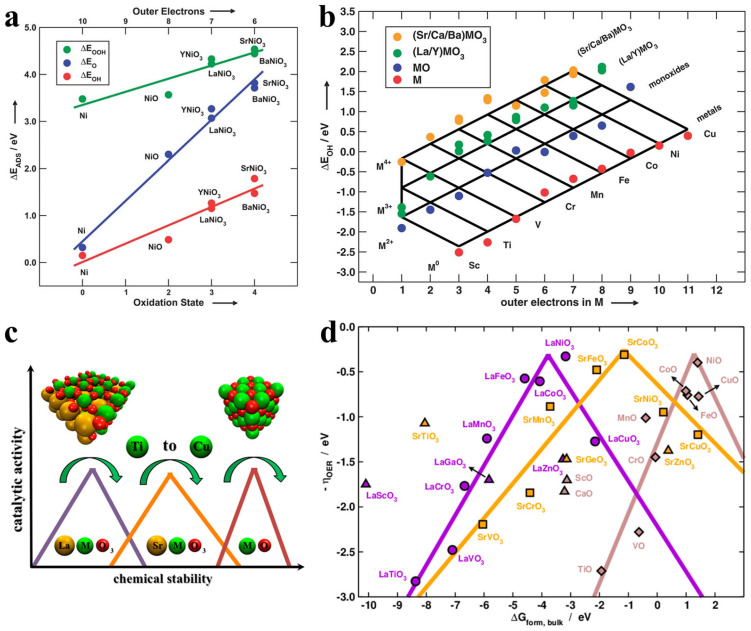
(**a**) Variations of the binding energy for different intermediates with the formula oxidation states in various compounds [47]. (**b**) The adsorption energy of *OH on various compounds with different outer electrons [47]. (**c**) The correlation of activity and stability in different compounds [49]. (**d**) Volcano-type activity plot for various compounds with different formation energy [49].

For transition metal-based electrocatalysts, the *d* orbital electron distribution of transition metals significantly influences the electronic structure. Understanding the relationship between the *d* electron density and OER activity is crucial for designing high-performance transition metal oxide catalysts. For simpler systems, the number of *d* electrons directly reflects the catalytic activity. According to Sabatier’s principle, the adsorption energy of reaction intermediates should neither be too strong nor too weak. Transition metal-based oxides, particularly Ni- and Co-based oxides, generally exhibit better OER catalytic activity [46]. Rossmeisl et al. systematically investigated the relationship between oxygen-based intermediates and the *d* electron count during the OER process (shown in Figure 3a,b) [47]. Theoretical calculations revealed that as the *d* electron count increases, the binding strength of intermediates also increases. A subsequent study indicated that the enhanced activity of transition metal oxides with different *d* electrons is related to increased occupancy of *d* antibonding orbitals and optimized binding strength of HO*, which endows Ni-based oxides with superior catalytic activity [48]. In addition to the OER activity, the chemical stability of oxide electrocatalysts also follows a volcano-like trend with the *d* electron count (shown in Figure 3c,d) [49]. The surface adsorption energetics and bulk thermochemistry depend similarly on the number of outer electrons of the transition metal in the oxide. In addition to the effect of the *d* electron on the catalytic activity, the interaction energies between metal centers and ligands play a crucial role. According to HSAB theory, hard acids prefer to bind with hard bases, while soft acids favor soft bases. Transition metals typically act as borderline acids, which can interact effectively with both hard and soft bases, depending on their electronic configuration and oxidation state [50]. The enhanced activity observed in certain transition metal oxides (e.g., Ru-based perovskites and pyrochlores) can be attributed to their ability to form strong interactions with oxygen ligands due to their intermediate hardness. These interactions facilitate optimal binding energies for catalytic reactions such as the HER and OER. Conversely, zinc-based complexes are generally considered hard acids, leading to weaker interactions with oxygen ligands, which are relatively harder bases. This mismatch results in less favorable binding energies, explaining why zinc-based complexes typically do not exhibit excellent catalytic activity in these reactions. In light of the key role of *d* orbital electrons in the optimization of reaction intermediate binding energies, these groundbreaking works provide significant guidance for the development of oxide electrocatalysts.

**Figure 4:**
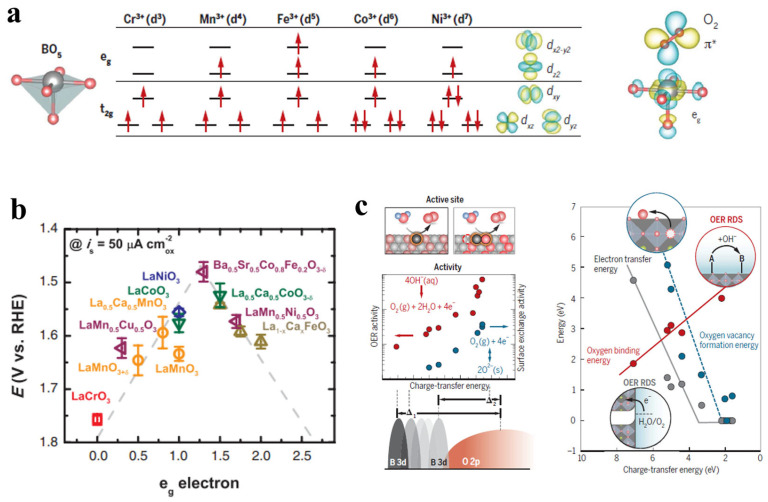
(**a**) Electronic configuration and relevant metal orbitals of first-row transition metals for a *B*O_5_ configuration [14]. (**b**) Relationship between overpotential of perovskite oxides and *e*_g_ orbital occupancy [52]. (**c**) Correlations between charge transfer energy (Δ) and OER activity [14].

Guided by molecular orbital theory, understanding the coordination environment of the metal–oxygen bond in the oxide lattice is crucial for tuning the electronic structure to regulate the surface binding energy. Taking first-row transition metals with octahedral coordination as examples (Figure 4a), the exposed *B*-sites have the coordination environment *B*O_5_ at the surface, breaking symmetry and leading to the splitting of *e*_g_ and *t*_2g_ degenerate orbitals into different energy levels [14]. According to frontier orbitals theory, the adsorbate intermediates would interact with the vertically oriented *e*_g_ orbital with stronger overlap than that with the *t*_2g_ orbital, thus determining the adsorption/desorption energy of the reaction intermediates. It should be noted that the spin states presented in Figure 4a are based on the experimental measurements of bulk LaMO_3_ (M = Cr, Mn, Fe, Co, Ni) materials at room temperature. Other configurations may also be observed under different conditions, such as varying temperatures or in thin-film geometries [51]. In fact, the spin states of the transition metal are largely dependent on the oxidation state and crystal structure (e.g., coordination environment, distortion), which should be determined by the magnetic measurements. Inspired by this point, Shao-Horn et al. systematically investigated the OER activity of a series of perovskite oxide electrocatalysts and proposed a significant *e*_g_-filling descriptor [52,53]. Based on *e*_g_ orbital occupancy, the *σ*-*e*_g_ orbital bonding has a stronger overlap with the oxygen-based reaction intermediates than that of the π-*t*_2g_ orbital bonding, directly influencing the binding energy of the adsorbates and promoting charge transfer, thereby enhancing OER performance. Therefore, as shown in Figure 4b, the occupancy of *e*_g_ orbitals correlates with the OER activity of perovskites exhibits a volcano-like relationship, in which the Ba_0.5_Sr_0.5_Co_0.8_Fe_0.2_O_3−*δ*_ (BSCF) at the peak of the volcano shows the best OER activity with an *e*_g_ filling of ~1.2. The *e*_g_-filling descriptor is also consistent with the Sabatier principle, where optimizing the binding strength between the reaction intermediates and the catalyst enhances the catalytic activity. Based on this theory, many outstanding OER electrocatalysts have been designed, such as CaCu_3_Ru_4_O_12_, Ca_2_Mn_2_O_5_, and CaCu_3_Fe_4_O_12_ [54,55,56]. To design an excellent OER oxide electrocatalyst with optimal *e*_g_ orbital occupancy, the *d* orbital can be modulated by regulating the oxidation state and spin state.

Although the *e*_g_-filling descriptor has been widely used in the electrocatalyst design, the available range of this descriptor is limited to explain the activity trends of all kinds of electrocatalysts. For example, the OER activity of the La*B*O_3_ (*B* = Cr, Mn, Fe, Co, Ni) system does not directly correlate with the *e*_g_ orbital occupancy [57]. Consequently, further theoretical and experimental studies have proposed more descriptors to establish catalytic-dependent models, such as metal–oxygen covalency and charge transfer energy [58,59]. For example, due to the sharing of electrons between the metal and oxygen atoms in highly covalent late-transition metal oxides, the metal and oxygen atoms can both be seen as active sites. To more precisely capture the mixed ionic–covalent character of transition metal oxides, metal–oxygen covalency derived from the bulk electronic structure has emerged as a powerful descriptor for OER theoretical studies. Specifically, the metal–oxygen covalency indicates the characters of metal *d*-orbitals and oxygen *p*-orbitals, dictating the stability and surface adsorption energetics [49,60,61]. Therefore, as shown in Figure 4c, the metal–oxygen covalency of oxides directly affects the oxygen binding energy, the oxygen vacancy formation energy, and the electron transfer barrier [14]. In detail, the enhanced metal–oxygen covalency would induce the O *p*-band center closer to the Fermi level, enhancing the charge transfer between the active site and reaction intermediates during the OER process, thus increasing the OER activity. Nevertheless, the stability of the oxide electrocatalysts would decrease with the increased metal–oxygen covalency; thus, the moderate metal–oxygen covalency would lead to both high activity and stability for the OER [58,59]. Therefore, metal–oxygen covalency can be used to predict and explain the OER activity of oxide electrocatalysts. For example, Xu et al. introduced Fe into LaCoO_3_, observing a transition from low-spin to high-spin Co in LaCo_0.9_Fe_0.1_O_3_, which increased the overlap between Co 3*d* and O 2*p* bands, enhancing the covalency and improving the OER performance [62]. Similar performance optimization can also be observed in spinel oxides such as ZnFe_x_Co_2−x_O_4_ [63]. In practice, doping the metal anion with different electronegative atoms or tuning the oxidation state of the metal anion can regulate the Fermi level and O 2*p* states, which can alter the metal–oxygen covalency and hybridization, consequently optimizing the OER activity of oxide electrocatalysts [64,65].

**Figure 5:**
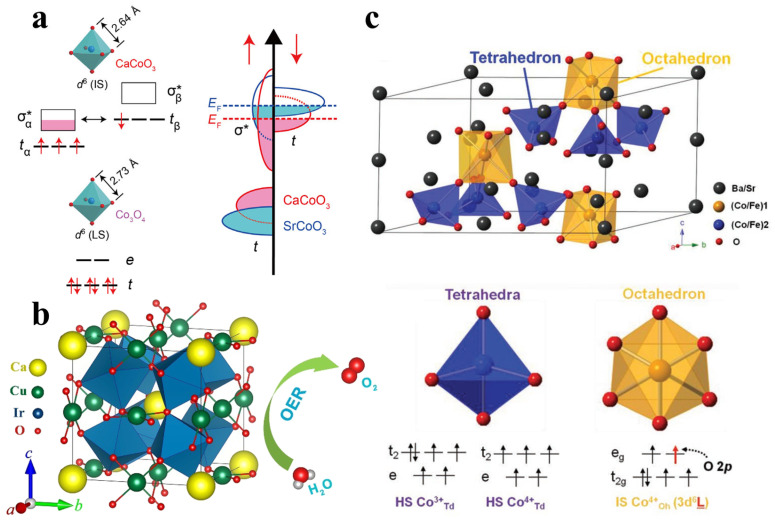
(**a**) Electronic structure changes in CaCoO_3_ and SrCoO_3_ [66]; (**b**) application of CaCu_3_Ir_4_O_12_ in OER [67]; (**c**) novel oxide OER catalyst Ba_4_Sr_4_(Co_0.8_Fe_0.2_)_4_O_15_ [68].

The discovery of new electrocatalysts is fundamental to investigating the structure–activity correlations, providing critical guidance for designing more efficient electrocatalysts and accelerating the development of practical applications. Based on advancements in theoretical and experimental studies, such as *e*_g_ orbital occupancy and metal–oxygen covalency, more novel oxide electrocatalysts have been developed for the OER. Goodenough et al. investigated the OER activity of the two isostructural *A*CoO_3_ (A = Ca, Sr) oxides synthesized via high-pressure synthesis [66]. As shown in Figure 5a, lattice parameter changes caused by different *A*-site ions led to modifications in the electronic structure of the oxides, resulting in superior catalytic activity for CaCoO_3_. Beyond simple perovskite oxides, quadruple perovskite has emerged as an efficient electrocatalytic system due to the complex electronic interaction between *A*- and *B*-site metal anions. For example, Long et al. synthesized a complex quadruple perovskite oxide CaCu_3_Ir_4_O_12_ for OER (shown in Figure 5b), which exhibited a low overpotential of 252 mV at 10 mA cm^−2^ in 1 M KOH, along with excellent long-term stability, demonstrating great potential for commercial applications [67]. In situ analysis revealed that the enhanced OER activity can be ascribed to the strong 3*d*–2*p*–5*d* orbital hybridization induced by the *A*’−*B* intersite cooperation. In addition, Shao et al. reported a novel oxide catalyst, Ba_4_Sr_4_(Co_0.8_Fe_0.2_)_4_O_15_, with a unique hexagonal structure. Both tetrahedral Co and octahedral O ions serve as OER active sites, resulting in exceptional OER activity (shown in Figure 5c) [68]. Additionally, many other outstanding oxide electrocatalysts (Table 1), such as Ba_2_*M*IrO_6_ (*M* = Y, La, Ce, Pr, Nd, and Tb) and *A*_2_Ru_2_O_7_ (*A* = Y, Nd, Gd, Bi), have been developed based on the above descriptors, where the development of new material systems has significantly advanced catalysis research, offering more electrocatalyst options for the OER [69,70]. Typically, pyrochlore oxides *A*_2_Ru_2_O_7_ (*A* = Y, Nd, Gd, Bi) are a class of promising OER catalysts with improved activity and stability compared to RuO_2_ [70]. The catalytic performance is influenced by the A-site cation, where a longer Ru–O bond and weaker Ru 4*d*–O 2*p* hybridization enhance the initial OER activity. Under acidic conditions, both A-site and Ru dissolution occur, affecting long-term stability. DFT-based Pourbaix diagrams confirm the thermodynamic instability of these materials, while theoretical predictions and Bader charge analysis suggest that dissolution exposes highly oxidized Ru sites with enhanced activity. Despite degradation, these pyrochlores exhibit significantly higher stability than RuO_2_, based on stability number metrics, offering a promising strategy for improving Ru-based catalysts in water electrolysis.

### 3.2. Overview of Bulk Oxide Electrocatalysts for Hydrogen Evolution Reaction

Due to the flexible composition and structures, bulk oxides have demonstrated significant performance advantages and application potential in the OER. In view of the excellent OER activity of oxides, it is of great demand to develop oxide electrocatalysts with highly efficient HER activity for practical applications of water electrolysis. However, the intrinsic disadvantages, such as poor electronic conductivity, inappropriate electronic structure for the HER, and limited active sites, have hindered the development of HER oxide electrocatalysts [79]. Therefore, due to the poor HER activity of bulk oxides, only a few oxides with high-performance HER electrocatalysts have been reported currently. To address this issue, substantial research has been dedicated to optimizing HER performance through different strategies, such as element doping, defect engineering, and morphology engineering [28,30,80]. To date, several bulk oxide systems have shown excellent HER activity that is even comparable to commercial Pt/C, indicating the promising potential for oxides in the HER. However, an ongoing debate still exists on the correlation between the intrinsic alkaline HER activity and electronic properties, which makes the design of bulk oxide catalysts for the HER largely a trial-and-error process.

**Figure 6:**
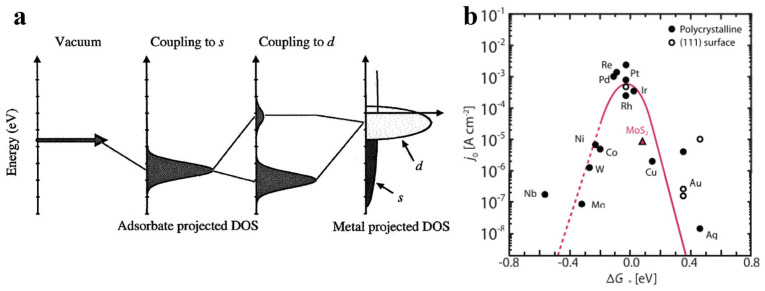
(**a**) The interaction between adsorbates and transition metal [85]. (**b**) Volcano plot of HER performance vs. Δ*G*_H*_ [22].

As mentioned above, the intermediate H* is included in all the pathways for the HER. Therefore, the Gibbs free energy change in hydrogen adsorption (Δ*G*_H*_) can theoretically evaluate the interaction between the H* intermediate and the active site, thereby determining the HER activity. In general, Δ*G*_H*_ for an ideal HER electrocatalyst should be close to zero value according to the Sabatier principle, which indicates the optimal absorption/desorption of H* on the catalyst surface. In addition, excellent HER electrocatalysts also enable a higher exchange current density. Therefore, by correlating experimental exchange current densities with theoretically calculated Δ*G*_H*_ values, a volcano diagram has been proposed to predict the activity [81,82,83]. Therein, Pt is located at the peak of the volcano with Δ*G*_H*_ close to 0, indicating that Pt is the most active HER electrocatalyst based on current research. An efficient HER electrocatalyst binds the reaction intermediate neither too strongly nor too weakly. Understanding how to regulate the binding energies of reactive intermediates on the catalyst surface is crucial for designing HER electrocatalysts with excellent activity. Based on the correlation between *d* electrons and catalytic performance, Nørskov proposed the *d*-band theory (also known as hydrogen binding energy, HBE), which analyzes the relationship between the *d*-orbital energy levels of transition metals and the adsorption energy of reaction intermediates [84,85,86]. The strength of the interaction between transition metals and reaction intermediates is determined by the filling of antibonding states, which is influenced by the energy of antibonding states relative to the Fermi level (shown in Figure 6a). Since antibonding states are always higher than *d* states, higher energy relative to the Fermi level results in stronger binding between intermediates and the catalyst. Thus, the energy of the *d*-state (relative to the Fermi level) can serve as an activity descriptor. The relative energy of the metal *d*-band and the antibonding hydrogen σ* orbital directly determines their overlap, which governs the bond strength of H intermediates to the metal surface (Δ*G*_H*_), explaining why Pt-based catalysts remain the best HER electrocatalysts (shown in Figure 6b) [22,87]. The *d*-band theory has been widely used to explain and design more efficient HER oxide electrocatalysts. Lotsch et al. investigated and compared the changes in HER performance of a class of delafossite oxides Pd*B*O_2_ (*B* = Cr, Co) and PtCoO_2_ under long-term electrocatalytic testing [88]. Through comprehensive analysis, the β-PdH_x_ layer formed in situ on the surface of PdCoO_2_ inherently stretched the Pd lattice, leading to the broadening of the *d*-band, and thus optimizing the adsorption energy of H*, consequently achieving catalytic performance comparable to commercial Pt/C catalysts.

Despite the *d*-band theory providing in-depth insight into the optimum catalyst for a given class of catalyst materials, there are additional factors that are needed to quantitatively determine the absolute reaction rates. Specifically, the kinetics barrier of the HER would change as a function of different pH conditions, leading to varying activity dependent on the pH values [89,90,91]. In acidic electrolytes, Δ*G*_H*_ can be used as a powerful descriptor to evaluate the HER activity, while in the alkaline or neutral electrolytes, the activation barrier of water dissociation and the binding hydroxyl intermediate would also influence the catalytic activity of the catalyst. Therefore, in addition to the previously mentioned Δ*G*_H*_, the Gibbs free energy change in hydroxyl desorption (Δ*G*_OH*_) and the kinetic barrier of water dissociation (*E*_b_) are jointly proposed as the descriptor for alkaline HER [92,93]. Water dissociation theory incorporates the kinetics of water dissociation and interfacial water/anion transfer, which can be ascribed to the H* originates from water dissociation under alkaline conditions [94]. Additionally, the adsorption of OH* competes with H* adsorption, affecting the utilization of active sites. Therefore, promoting OH* desorption or providing OH* adsorption sites can enhance H* generation and utilization, thereby improving the overall HER efficiency [95,96]. Recently, more specific HER descriptors have been proposed for the understanding of the HER process, such as zero-charge potential theory and a hydrogen bond network [97,98]. Zero-charge potential theory suggests that the surface double layer of the catalyst changes with the pH, controlling the binding of reaction intermediates H* and OH* on the catalyst surface, providing some explanation for the “dual-site” catalytic mechanism [99]. Nevertheless, no single descriptor can universally explain the experimental phenomena, and multiple theories are often needed to deepen the understanding of the structure–activity correlations for the HER. Therefore, further theoretical research is required to build effective explanatory models and guide the discovery and design of more efficient electrocatalysts.

**Figure 7:**
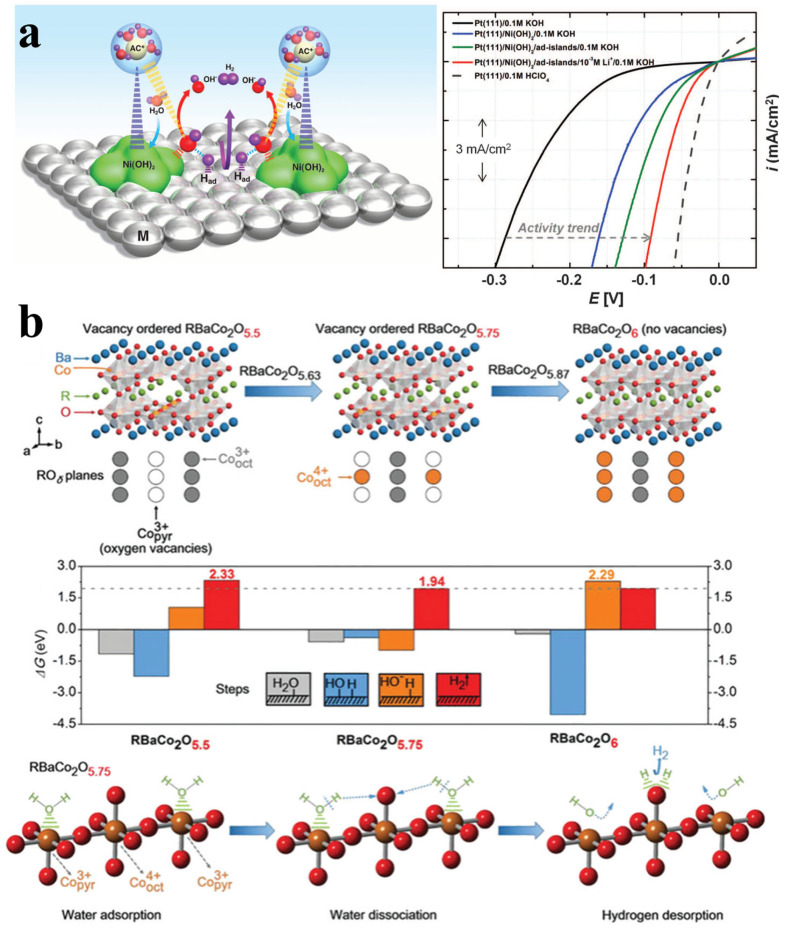
(**a**) Schematic of alkaline HER based on water dissociation theory [94]. (**b**) Relationship between HER performance of *R*BaCo_2_O_5.5+δ_ and oxygen vacancies [100].

Based on advancements in theoretical and experimental studies, such as *d*-band theory, more novel oxide electrocatalysts have been developed for the HER (Table 2). In view of the considerable OER activity of oxides, it is expected that oxide electrocatalysts may enable significant potential for water dissociation in the HER process. For example, Markovic et al. reported a Ni(OH)_2_-Pt composite HER electrocatalyst with a factor of 8 activity increase in HER activity relative to state-of-the-art catalysts (Figure 7a) [94]. The enhanced HER performance can be attributed to the dissociation of water promoted by Ni(OH)_2_ clusters. In addition, as shown in Figure 7b, Shao et al. systematically investigated the alkaline HER performance of *A*-site-ordered double perovskite oxides *R*BaCo_2_O_5.5+δ_ (R = lanthanides) [100]. Therein, the synergistic effects of high-spin Co^3+^ tetrahedral oxygen vacancies and intermediate-spin Co^4+^ octahedral vacancies in *R*BaCo_2_O_5.75_ could enhance H_2_O adsorption and dissociation accompanied by the optimization of H intermediate adsorption, ultimately improving the overall HER activity. Later, Shao et al. explored the influence of different *A*-site ions on the performance of *R*BaCo_2_O_5.5+δ_, suggesting that A-site ion electronegativity can serve as a descriptor for HER activity prediction and evaluation [101]. Theoretical calculations of 13 different *R*BaCo_2_O_5.5+δ_ compositions revealed that the *A*-site ion electronegativity plays a key role in determining the electronic structure of *B*-site ions, where Gd_0.5_La_0.5_BaCo_2_O_5.5+δ_ (electronegativity~2.33) exhibits the best HER activity due to the optimal electronic structure. The flexible crystal structure and composition of perovskite oxides have facilitated the emergence of more oxide-based HER electrocatalysts, such as SrRuO_3_, Sr_2_RuO_4_, BaRuO_3_, and so on [102,103,104]. Notably, the Ruddlesden–Popper-type layered oxide Sr_2_RuO_4_ exhibits outstanding HER activity in alkaline media, comparable to the best electrocatalysts reported to date [104]. Theoretical calculations reveal that this performance stems from an unusual synergistic effect between its perovskite and rock-salt layers—the SrO-terminated (001) surface enables barrier-free water dissociation, while apical oxygen sites in the perovskite layer facilitate favorable hydrogen adsorption and evolution. Therefore, designing and identifying more high-performance oxide HER electrocatalysts through the combination of theory and experiments is crucial for designing and developing more efficient HER electrocatalysts.

## 4. Design Strategy for Bulk Oxides with High Water Electrolysis Performance

The catalyst design has been the research focus on electrochemical reactions that promise energy conversions and storage. Great attention, for example, was dedicated to developing earth-abundant transition metal-based nanomaterials, aiming to achieve high catalytic performance at a low cost. Unfortunately, nanocatalysts typically suffer from unsatisfactory repeatability, especially the lack of a scaled-up synthesis strategy for industrial manufacture, thereby impeding their commercial potential. Therefore, research efforts on catalyst design are still urgently demanded for electrocatalysis. Bulk oxides address some critical aspects of water electrolysis (especially scaled-up synthesis using solid-state reactions), but they still have inherent drawbacks, such as unsatisfactory activity and severely limited performance by virtue of the finite active sites in the bulk materials. To further enhance the performance and selectivity of bulk oxide electrocatalysts, it is essential to design and develop more advanced electrocatalysts. Currently, there are generally two strategies to improve the activity of an electrocatalyst: increasing the number of active sites and enhancing the intrinsic activity of each site [22]. In fact, increasing the number of active sites and enhancing the intrinsic activity often occur simultaneously. Different design strategies can be tailored based on these principles to achieve superior catalytic activity.

### 4.1. Crystal Structure Engineering

**Figure 8:**
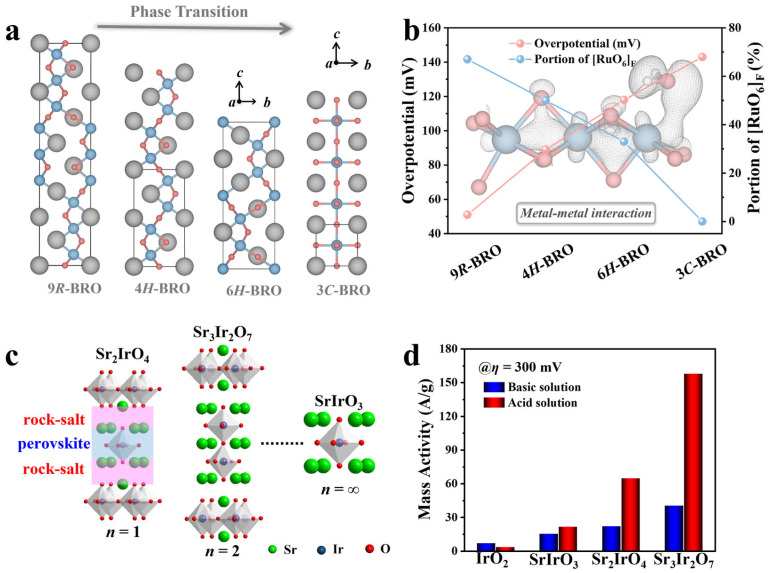
(**a**) Pressure-dependent structural transition of BaRuO_3_ from 9*R*- to 4*H*-, 6*H*-, and 3*C*- polymorphs [7]. (**b**) Variation in overpotential at 10 mA cm^−2^ reflecting different proportions of face-shared octahedra in BRO polymorphs [7]. (**c**) Crystal structures of Sr*_n_*_+1_Ir*_n_*O_3*n*+1_ (*n* = 1, 2, and ∞) [75]. (**d**) Mass activity of IrO_2_ and Sr*_n_*_+1_Ir*_n_*O_3*n*+1_ (*n* = 1, 2, and ∞) at the overpotential of *η* = 300 mV in acid and basic solutions [75].

Exploring a relatively simple and clear system to realize intrinsic activity regulation is urgent and significant for oxide catalysts. Structural phase transitions provide a treasure route to intensify the systematic understanding of the correlation between the electrocatalytic activity and the crystal, as well as the electronic structure of a material, such as noble metal and transition metal dichalcogenides [111,112,113]. Altering interatomic distances and bonding patterns without any composition change is expected to alter the electronic structure and thus adjust the catalytic performance. In our recent study, the model system barium ruthenate (BaRuO_3_, BRO) with four distinct polymorphs is synthesized by using the solid-state reaction and high-pressure/high-temperature (HPHT) method to rationalize the impact of the crystal as well as electronic structure on the HER activity (Figure 8a) [7]. As a micron-sized oxide, the BRO in the 9*R* phase (a rhombohedral stacking variant with nine layers per unit cell) displays an exceptional alkaline HER activity comparable to commercial Pt/C (Figure 8b). The substantial electronic configuration is revealed by the combination of synchrotron X-ray analytical techniques, magnetic and electric measurements, and density function theory calculations. The direct *d-d* hopping in face-shared RuO_6_ octahedra leads to strong orbital overlap with the H_2_O molecule, which renders fast kinetics of water splitting and optimizes the binding energy with surface reactants, thus tremendously improving the HER efficiency of the bulk 9*R*-BRO. More importantly, 9*R*-BRO can be easily synthesized on a large scale using solid-state reactions, showing reproducible catalytic activity and superior transferability in commercial alkaline water electrolyzers, signifying the great potential of bulk oxide electrocatalysts in practical applications. This work highlights the influence of the crystal structure on the activity of oxide electrocatalysts, suggesting phase transition is a valid design principle for advanced electrocatalysts with rational activity enhancement.

In addition to the structural phase transition, the dimensionality control of the crystal structure can also boost the catalytic activity of the oxide electrocatalysts. The layered Ruddlesden−Popper (R-P) oxides have emerged as a more promising OER catalyst family due to the flexible perovskite layers in the lattice [16,114,115]. Previous studies have shown that the OER performances of R-P oxides are largely associated with the inherent dimensionality arising from the different perovskite layers. For example, the OER performances are investigated for a series of R-P oxides La*_n_*SrNi*_n_*O_3*n*+1_ (*n* = 1, 2, 3 and ∞), showing the OER activity enhanced with the increasing perovskite layers *n* [116]. More theoretical and experimental results indicate that the optimized activity of the R-P nickelates can be ascribed to the insulator-to-metal transition and strengthened Ni-O hybridization induced by the increased dimensionality. It is worth noting that the trends of the OER activity are also associated with the composition. In our recent study (Figure 8c,d), we sought to intensify the systematic understanding of the intrinsic effect of the number of perovskite layers in the OER by investigating a prototypical family of R-P iridate oxides Sr*_n_*_+1_Ir*_n_*O_3*n*+1_ (*n* = 1, 2, and ∞) [75]. Unveiled by experiments and theoretical calculations, both the conductivity and binding energy of oxygen intermediates on the metal site depend on the number of perovskite layers, resulting in the enhanced OER intrinsic activity of Sr_3_Ir_2_O_7_. These studies provide enticing strategies for developing advanced electrocatalysts via unique crystal structure designs in renewable energy applications.

### 4.2. Heteroatom Doping

**Figure 9:**
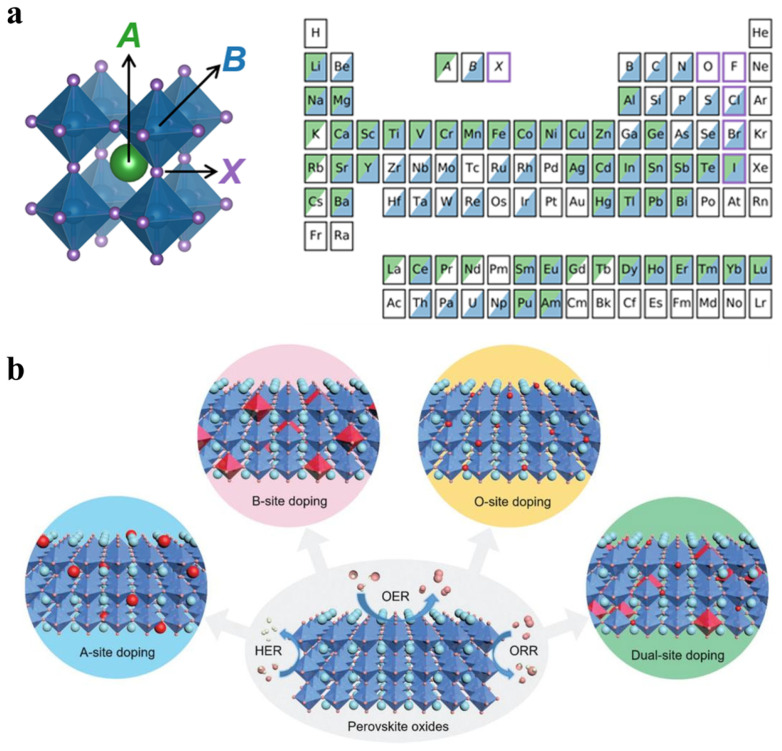
(**a**) The typical perovskite structure and composition [100]. (**b**) Element doping strategies at different sites of perovskite oxides [101].

The electronic structure plays a vital role in determining the catalytic activity of electrocatalysts, where heteroatom doping has been broadly used to modulate the electronic structure, like the *d*-band center, consequently altering the water electrolysis performance. Taking perovskite oxides as an example, as shown in Figure 9, over 90% of periodic table elements can be incorporated into the perovskite structures [117]. By replacing elements at different crystallographic positions in perovskite oxides (*AB*O_3_), both the *A*- and *B*-site, as well as O-site, the electronic structure can be tuned to optimize the catalytic activity due to the different ionic radius and electronic structure from the heteroatom [118]. Typically, *A*-site ions are composed of alkali, alkaline earth, or rare-earth metals, which cannot directly participate in catalytic reactions. However, the heteroatom doping at the *A*-site would induce the change in the crystal structure, such as the contraction or expansion of the lattice parameters, structural distortions, and phase changes, and thus alter the electronic structure of *B*-site ions to affect the catalytic performance. For example, Müller et al. investigated the impact of different *A*-site ion doping on the OER performance of pyrochlore Y_1.8_M_0.2_Ru_2_O_7−*δ*_ (*M* = Cu, Co, Ni, Fe, Y). Different *A*-site ions can influence the surface lattice oxygen defect concentration and alter the energy difference between the O 2*p* band and the Fermi level (EF), thereby modulating the intrinsic OER activity [119]. In addition, the introduction of heterovalent atoms can also alter the oxidation state of *B*-site ions, like the enhanced OER stability of SrRuO_3_ by Na doping, which can be ascribed to the increased Ru oxidation states and the distortion of RuO_6_ octahedra [120].

*B*-site ion doping can directly modify the electronic structure of active sites, thus enhancing the electrocatalytic activity of electrocatalysts. For example, Lu et al. studied the effects of Co ion doping on the layered perovskite oxide Bi_7_Fe_3_Ti_3_O_21_ [121]. The elongation of oxygen octahedra in the layered structure can stabilize the intermediate-spin Co^3+^ state, providing an optimal electronic configuration for OER. Additionally, Co doping improved the conductivity of the oxide and optimized oxygen intermediate adsorption, resulting in efficient OER performance. More importantly, diluting the content of precious metal in the crystal structure is a sought-after strategy for designing efficient electrocatalysts. For instance, perovskite oxide SrIrO_3_ has been demonstrated as a highly active electrocatalyst exceeding the benchmark OER catalysts such as IrO_2_ and RuO_2_ [72]. Further introduction of Zr or Ti at the Ir-site not only decreased the content of iridium, but also enhanced the intrinsic OER activity more than that of SrIrO_3_, confirming the great advantages of the heteroatom doping strategy [122,123]. In addition to cation doping, anion doping is another effective strategy to improve the catalytic activity, such as the F, S, N, or halogen elements at O-sites. Due to the discrepancy of electronegativity and valency, these dopants can alter the oxygen vacancy formation energy, adjust the electronic structure, and induce surface structural changes, thereby influencing the overall catalytic performance. Therein, F atom doping is widely used in transition metal oxides due to the inherently most electronegative anion with a −1 charge. For example, F anion doping in La_0.5_Ba_0.25_Sr_0.25_CoO_2.9−δ_F_0.1_ could alter the CoO_6_ coordination environment, which induced CoO_6_ octahedra to transform into CoO_5_F octahedra and CoO_5_ pyramids, thus promoting Co *e*_g_ and *t*_2g_ orbital electron rearrangement [124]. Therefore, the introduction of the F anion resulted in the upshift of the O-*p* band center and activated the lattice O in the OER process, consequently boosting the OER activity for oxides.

### 4.3. Defect Engineering

In addition to heteroatom doping, defect engineering is also an effective strategy to alter the electronic structure without additional elements being introduced into the lattice, providing a relatively simple platform to uncover the activity–structure correlations [125]. Commonly, defects can be introduced into the cation and anion sublattices as vacancies or interstitials, respectively [126]. Thus, defect engineering can greatly tailor the physicochemical properties and electronic structure, consequently optimizing the performances. Anion defects have been extensively investigated in oxide electrocatalysts, which can be introduced through various methods, including a topochemical reduction reaction, heterovalent ion doping, etc. For example, Kim et al. synthesized single-phase Ca_2_Mn_2_O_5_ by a reduction reaction under 5% H_2_/Ar atmosphere, successfully introducing oxygen vacancies in the lattice and thus accelerating charge transfer during the OER [56]. Compared to the original CaMnO_3_, Ca_2_Mn_2_O_5_ exhibited a 100 mV lower onset potential and 4–6 times higher mass activity, which can be attributed to the regulation of Mn *d* orbital electron occupancy induced by oxygen vacancy and faster OH^−^ transfer in the OER process. In addition, theoretical studies also found that oxygen vacancy formation energy correlates with the energy difference between the O 2*p* band center and *E*_F_, where the lower O 2*p* band center indicates the higher oxygen vacancy formation energies [64]. For example, Ciucci et al. synthesized layered NdBaMn_2_O_5.5_, where ordered oxygen vacancies occupied the apex of MnO_5_ pyramids, alternating with MnO_6_ octahedra along the *b* axis [127]. The introduction of oxygen vacancies optimized the Mn *d* orbital alignment and O 2*p* band center with the *e*_g_ orbital electron occupancy~1.04, consequently improving charge transfer and optimizing the adsorption energy between the catalysts and reaction intermediates. Therefore, at an applied voltage of 1.7 V, NdBaMn_2_O_5.5_ exhibited 24 times higher intrinsic activity and 2.5 times higher mass activity than the commercial RuO_2_ catalyst for the OER.

In recent years, there has been growing interest in cation defect engineering to optimize oxide catalytic performance [128]. Cation defects can directly adjust the electronic structure of active sites, such as regulating the *d* orbital electron distribution of transition metals, thereby influencing the catalytic activity. Additionally, *A*-site defects can modify the oxidation state of B-site ions, which is accompanied by increased oxygen vacancies in the lattice. For example, Shao et al. synthesized a series of *A*-site-deficient La_1−*x*_FeO_3−*δ*_, which exhibit enhanced OER and ORR activities than those in LaFeO_3_ [129]. Structural characterization revealed that La vacancies promoted the formation of Fe_4+_ and surface oxygen vacancies, significantly improving the catalytic activity. In addition, *A*-site defects can also optimize the electronics of *B*-site ions, such as *e*_g_ orbital filling, further enhancing the OER activity. For example, Gong et al. synthesized *A*-site-deficient Sr_x_Co_0.8_Fe_0.2_O_3−*δ*_ with enhanced OER performance under alkaline conditions, which can be ascribed to the optimization of *e*_g_ occupancy of active sites [130]. Nevertheless, undesired defects would also result in unexpected performance reduction. Thus, defect engineering requires precise control over the synthesis condition.

### 4.4. Morphology Engineering

**Figure 10:**
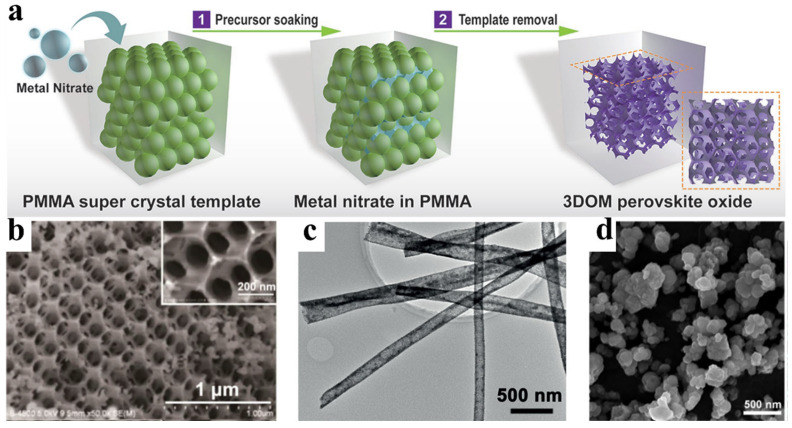
(**a**,**b**) The 3D porous LaFeO_3_ synthesized via templating [133]; (**c**) SrTi(Ir)O_3_ nanofibers [134]; (**d**) P-Tl_2_Ru_2_O_7_ nanoparticles [135].

In addition to enhancing the intrinsic activity mentioned in the above strategies, regulating the morphology of oxide electrocatalysts is an effective strategy to improve catalyst performance. Bulk oxides are typically synthesized via high-temperature solid-state reactions, resulting in micrometer-sized particles with low specific surface areas and limited active sites. Engineering the morphology of bulk oxides, such as the porous structure, nanostructure, and heterostructure, can increase the number of exposed active sites and thus boost the performance of electrocatalysts [27,131,132]. For example, Shao et al. synthesized a three-dimensional ordered microporous (3DOM) LaFeO_3_ catalyst via the colloidal crystal template method [133]. As shown in Figure 10a,b, compared with bulk LaFeO_3_, the 3DOM-LaFeO_3_ catalyst exhibited an enhanced specific surface area and active site exposure, boosting the activity and stability markedly for the OER and HER. Additionally, Zou et al. fabricated porous SrTi(Ir)O_3_ nanotubes by electrospinning methods (as shown in Figure 10c) [134]. Specifically, compared to SrTi(Ir)O_3_ nanoparticles, the porous nanotubes exhibited a 2.7 times higher specific surface area and superior OER activity in acidic conditions. However, due to the limitation of high temperatures in synthesizing the mixed oxides, only limited oxides have been fabricated in the nanostructure. The design of well-defined nanostructures plays a crucial role in enhancing the electrocatalytic performance of oxide materials, as it can significantly increase the surface area, expose more active sites, and modulate the electronic structure. Alternatively, the sol–gel method offers a promising route for synthesizing oxide materials with well-controlled nanoparticle structures. For instance, Kim et al. successfully synthesized pyrochlore-type Tl_2_Ru_2_O_7_ nanoparticles (~200 nm, Figure 10d) using a sol–gel approach, followed by surface functionalization with dihydrogen phosphate groups to yield P-Tl_2_Ru_2_O_7_ nanoparticles [135]. The resulting P-Tl_2_Ru_2_O_7_ catalyst exhibited excellent bifunctional activity toward both the oxygen evolution reaction (OER) and the oxygen reduction reaction (ORR), enabling high-performance air cathodes in hybrid Na–air batteries. The enhanced catalytic performance can be attributed to two key factors: (1) the increased Ru–O bond covalency induced by surface dihydrogen phosphate functionalization, and (2) the significantly enhanced specific surface area arising from the nanoparticulate structure.

## 5. Conclusions and Perspective

Due to the advantages of flexible composition and structure, substantial development has been achieved in bulk oxide catalysts, providing a good platform for studying the correlation between the activity and structure. For instance, perovskite oxides such as 9*R*-BaRuO_3_ demonstrate approachable scaled-up synthesis and efficient alkaline water electrolyzer performance [7]. Moreover, pyrochlore oxides *A*_2_Ru_2_O_7_ (*A* = Y, Nd, Gd, Bi) represent a promising class of OER catalysts, showing not only enhanced catalytic activity but also improved durability compared to RuO_2_, as evidenced by lower Tafel slopes and higher stability numbers [70]. In this review, we have introduced the recent breakthroughs in bulk oxide electrocatalysts for water electrolysis, especially for the development of main descriptors describing the activity dependence, such as the *e*_g_ orbital occupancy, *d*-band theory, etc. Although bulk oxides address the critical aspects of electrocatalysts (scaled-up synthesis using solid-state reactions), their inherent limitations, such as the finite active sites associated with the bulk nature, led to slightly inferior performance compared to nanocatalysts. To address the limitation of performance, several strategies are highlighted in this review, including optimizing the crystal/electronic structures and increasing the active sites. Although bulk oxides have demonstrated excellent water electrolysis performance in extensively researched studies, several limitations and challenges are still remaining in the current field. From our point of view, the bulk oxides for water electrolysis that need to be further investigated are schematically shown in the following aspects:

### 5.1. Developing New Electrocatalysts

While some bulk oxides have achieved significant developments in water electrolysis, the discovery of new bulk oxide electrocatalysts with better activity remains a hot spot in this field. To date, only a limited number of oxides have been explored for water electrolysis. Advances in solid-state chemistry have enabled the synthesis of a vast array of new oxide materials, with over 90% of the elements in the periodic table being potentially incorporated into their compositions and structures. Furthermore, the development of high-pressure synthesis techniques has significantly expanded the diversity of available bulk oxides. As a result, the extensive chemical space offered by these materials holds great promise for the design of novel electrocatalysts with superior performance. Up to now, only a few oxides have been investigated for water electrolysis, and thus, more corresponding research is invited to expand the variety of electrocatalysts. In addition, most bulk oxide electrocatalysts are typically micron-sized due to the limitations of widely used solid-state reactions (e.g., high temperature, long reaction time). Only limited classes can be synthesized in nanosized morphology using moderate strategies. Although the bulk oxides with micron-sized morphology have demonstrated stability under industrial plasma spraying for the electrode fabrication of AWE, developing nanostructured oxide electrocatalysts remains the focus of research of water electrolyzer techniques, like PEMWE. The bulk nature not only results in finite active sites for catalytic reactions, but also impedes the further preparation of a homogeneous catalyst layer in membrane-based water electrolyzer techniques. It is, therefore, a standing challenge to realize the synthesis of bulk oxide electrocatalysts in nanostructured morphology using moderate strategies.

### 5.2. Deepening the Understanding of Structure–Activity Correlations

The correlation between structure and activity is crucial for the design of efficient electrocatalysts. Although an in-depth understanding of water electrolysis has been achieved for bulk oxides, the intrinsic characteristic dominating activity and stability remains elusive. For example, the main reported descriptors, like the *d*-band center, are usually based on a linear scaling relationship (e.g., volcano plot), which has the upper limitation for optimizing performance. In addition, the available range of each descriptor is limited in predicting the activity trends of all kinds of electrocatalysts. Therefore, the development of more universal and comprehensive descriptors is vital for future research on structure–activity correlations. Moreover, identifying precisely the fundamental structures (crystal/electronic/surface structure) of the electrocatalysts is the premise for investigating the structure–activity correlations. To unravel the intrinsic activity dependence, combining integrated theoretical calculations and advanced characterization techniques (especially in situ analysis) would be an accessible way. Recently, emerging factors that may significantly influence the catalytic performance have attracted increasing attention. For example, BaRuO_3_ exhibits four distinct crystalline phases with markedly different HER activities, suggesting that the connectivity modes of octahedra in perovskite structures—such as face-sharing, edge-sharing, or corner-sharing configurations—can play a critical role in determining the catalytic performance. Furthermore, recent studies indicate that noble metals such as Ir in higher oxidation states may exhibit enhanced catalytic activity. However, it remains to be determined whether this phenomenon is observed in just a few specific instances or represents a broader trend, and what underlying mechanisms are responsible.

### 5.3. Correlation of Atomic Properties of Transition Metals with Ligand Interactions

A profound understanding of the atomic characteristics of transition metals, including electronegativity, ionic radius, and *d*-orbital energy levels, is crucial for elucidating their potential interactions with ligands. These intrinsic properties significantly influence the binding affinity, electronic structure, and catalytic behavior of the metal centers. Despite numerous studies exploring transition metal complexes, most investigations tend to treat these atomic features separately or focus primarily on one aspect, which can overlook the complex, combined effects these properties have on coordination behavior and reactivity. Therefore, future research efforts should emphasize multi-parameter analyses that simultaneously consider these atomic factors, supported by both experimental data and theoretical modeling. Such integrated studies will fill a key gap in the field, enhancing our ability to tailor transition metal complexes for specific applications more effectively.

### 5.4. Practical Applications for Water Electrolysis

Despite decades of research, substantial advancements in efficient electrocatalyst design have been achieved on the laboratory scale, while these have not improved the urgent requirements of industrial water electrolyzer performance. Bridging the gap between advanced electrocatalysts and highly effective electrodes in the device is one of the main challenges in achieving cheap hydrogen. Therefore, bulk oxide electrocatalysts should be evaluated in real water electrolyzers in future research. In addition, comprehensive improvements in activity and stability under industrial conditions are another key target for practical applications (e.g., high current densities, long operating time, high temperature). Still, the advanced electrocatalysts in the laboratory fall short of meeting the demand for commercial devices. Therefore, it is imperative to develop robust electrodes with efficient electrocatalysts to ensure the long-term stability of water electrolyzers.

## Figures and Tables

**Table 1:** Summary of representative bulk oxide electrocatalysts for OER.

Catalysts	Electrolyte	*η* at 10 mA cm^−2^ (mV)	Tafel Slope (mV dec^−1^)	Reference
Ba_2_*M*IrO_6_ (*M* = Y, La, Ce, Pr, Nd, Tb)	0.1 M HClO_4_	>370	60–120	[69]
Y_2_Ir_2_O_7_	0.1 M HClO_4_	262	50	[71]
6*H*-SrIrO_3_	0.5 M H_2_SO_4_	248	/	[72]
CaCu_3_Ru_4_O_12_	0.5 M H_2_SO_4_	171	40	[54]
Ba_3_TiIr_2_O_9_	0.1 M HClO_4_	275	45.7	[73]
Ba_4_Sr_4_(Co_0.8_Fe_0.2_)_4_O_15_	0.1 M KOH	340	47	[68]
Sr_2_*M*IrO_6_ (*M* = Ni, Co, Sc, Fe)	0.1 M HClO_4_	295–420	48–90	[74]
CaCu_3_Ir_4_O_12_	1 M KOH	252	47	[67]
Sr_3_Ir_2_O_7_	0.5 M H_2_SO_4_	259	50	[75]
Cu_2_IrO_3_	1 M KOH	361	51	[35]
SrIr_2_O_6_	0.1 M HClO_4_	303	44.2	[76]
Dy_2_NiRuO_6_	0.1 M HClO_4_	277	58	[77]
Li_2_Mn_0.85_Ru_0.15_O_3_	0.1 M KOH	260	49.6	[78]

**Table 2:** Summary of representative bulk oxide electrocatalysts for HER.

Catalysts	Electrolyte	*η* at 10 mA cm^−2^ (mV)	Tafel Slope (mV dec^−1^)	Reference
Pr_0.5_(Ba_0.5_Sr_0.5_)_0.5_Co_0.8_Fe_0.2_O_3−*δ*_	1 M KOH	237	45	[105]
(Gd_0.5_La_0.5_)BaCo_2_O_5.5+*δ*_	1 M KOH	210	27.6	[101]
PrBaCo_2_O_5.5+*δ*_	0.1 M KOH	291	89	[106]
SrRuO_3_	1 M KOH	101	67	[104]
Sr_2_RuO_4_	1 M KOH	61	51	[104]
SrRu_0.9_Co_0.1_O_3−*x*_	1 M KOH	57.8	35	[102]
Sr_4_Ru_2_O_9_	1 M KOH	28	55	[107]
SrTi_0.7_Ru_0.3_O_3−*x*_	1 M KOH	46	40	[108]
BaMoO_3_	1 M KOH	336	110	[109]
9*R*-BaRuO_3_	1 M KOH	51	30	[7]
La_2_Sr_2_PtO_7+*δ*_	0.5 M H_2_SO_4_	13	22	[110]

## Data Availability

Source data are available from the corresponding authors upon reasonable request.
